# Development of potent promoters that drive the efficient expression of genes in apple protoplasts

**DOI:** 10.1038/s41438-021-00646-4

**Published:** 2021-10-01

**Authors:** Xianpu Wang, Lili Xu, Xiuxia Liu, Li Xin, Shujing Wu, Xuesen Chen

**Affiliations:** College of Horticultural Science and Engineering, State Key Laboratory of Crop Biology, Shandong Agricultural University, Tai’an, Shandong PR China

**Keywords:** Non-model organisms, Pattern recognition receptors in plants

## Abstract

Protoplast transient expression is a powerful strategy for gene functional characterization, especially in biochemical mechanism studies. We herein developed a highly efficient transient expression system for apple protoplasts. The abilities of the *Arabidopsis thaliana* and *Malus domestica ubiquitin-10* (*AtUBQ10* and *MdUBQ10*) promoters to drive the expression of multiple genes were compared with that of the *CaMV 35S* promoter, and the results revealed that the *AtUBQ10* and *MdUBQ10* promoters were more efficient in apple protoplasts. With this system, we demonstrated that active AtMKK7ac could activate MAPK6/3/4 signaling cascades, which further regulated MdWRKY33 phosphorylation and stability in apple. Furthermore, the ligand-induced interaction between the immune receptor AtFLS2 and the coreceptor AtBAK1 was reconstituted in apple protoplasts. We also found that the stability of the bacterial effector AvrRpt2 was regulated by feedback involving auxin and the immune regulator RIN4. The system established herein will serve as a useful tool for the molecular and biochemical analyses of apple genes.

## Introduction

Apple is a rosaceous fruit tree plant that is cultivated worldwide, and researchers are becoming increasingly interested in the identification of the functionally important genes and molecular mechanisms involved in controlling apple fruit and plant growth and development^1–3^, fruit quality^4–8^, resistance^9–12^, and other physiological activities^13,14^. The expression of genes in plant materials is of critical importance for gene functional characterization and signal transduction pathway identification. Currently, apple genes are transiently expressed in onion epidermal cells^14^, apple and maize protoplasts^7,8,13,14^, pollen tubes^13^, tobacco and apple leaves^4,10^ and apple fruit surface cells^5^ and are stably expressed in transgenic apple plants^3,7,12^, callus^6–8,11^ and *Arabidopsis*^8^ for gene functional characterization. A protocol for transient expression in apple fruit cells was also developed by Spolaore et al.^15^. All these methods contribute to elucidating the molecular mechanisms controlling the physiological processes in apple.

Protoplasts are cell wall-free cells isolated from plant tissues and are capable of perceiving external stimuli. Transient expression in protoplasts has been proven to be a powerful strategy for gene functional characterization and signal pathway identification^16^, as it provides valuable information for understanding the molecular mechanisms controlling plant immune responses, hormone signaling, growth and development, epigenetic gene expression regulation and other physiological processes^17–20^. The expression of target genes in protoplast cells can be suppressed by the overexpression of artificial microRNAs or by modulation of their regulatory elements^20,21^. Mutagenesis in protoplast cells can be performed by CRISPR/Cas9 gene editing^22,23^. Protoplast transient expression has been found to efficiently, precisely, accurately, and consistently reveal molecular mechanisms. In apple, the use of an apple protoplast transient expression strategy is of special and critical importance. It can realize homologous expression without technical obstacles or the long time required to obtain transgenic apple plants and callus. Importantly, it is unaffected by the problems of heterologous expression. For example, certain *Arabidopsis* proteins were mislocalized after their expression in tobacco^24^. A precisely controlled expression strategy with an accurate evaluation method will be a powerful tool for apple gene functional characterization and signal transduction pathway identification, which will substantially deepen the understanding of the gene functions and signaling processes involved. In a recent report, apple protoplasts were subjected to CRISPR/Cas9 modification to generate genome-edited apple plants^23^. Despite the application of this method in several studies on subcellular localization and protein interactions^7,8,13^, it has not been extensively employed in studies on apple gene functional characterization and signal transduction pathway identification, especially in biochemical mechanism studies. Low gene expression in apple protoplast cells might be an important factor impeding the use of apple protoplasts.

In this study, target genes were stably and efficiently expressed in apple protoplasts by using the *AtUBQ10* or *MdUBQ10* promoter. The data obtained herein using these promoters provide insight into the novel mechanisms regulating immune and disease resistance in apple. This research paves the way for the application of an apple protoplast transient expression strategy to elucidate biochemical mechanisms in the rosaceous plants.

## Results and discussion

**Fig. 1:**
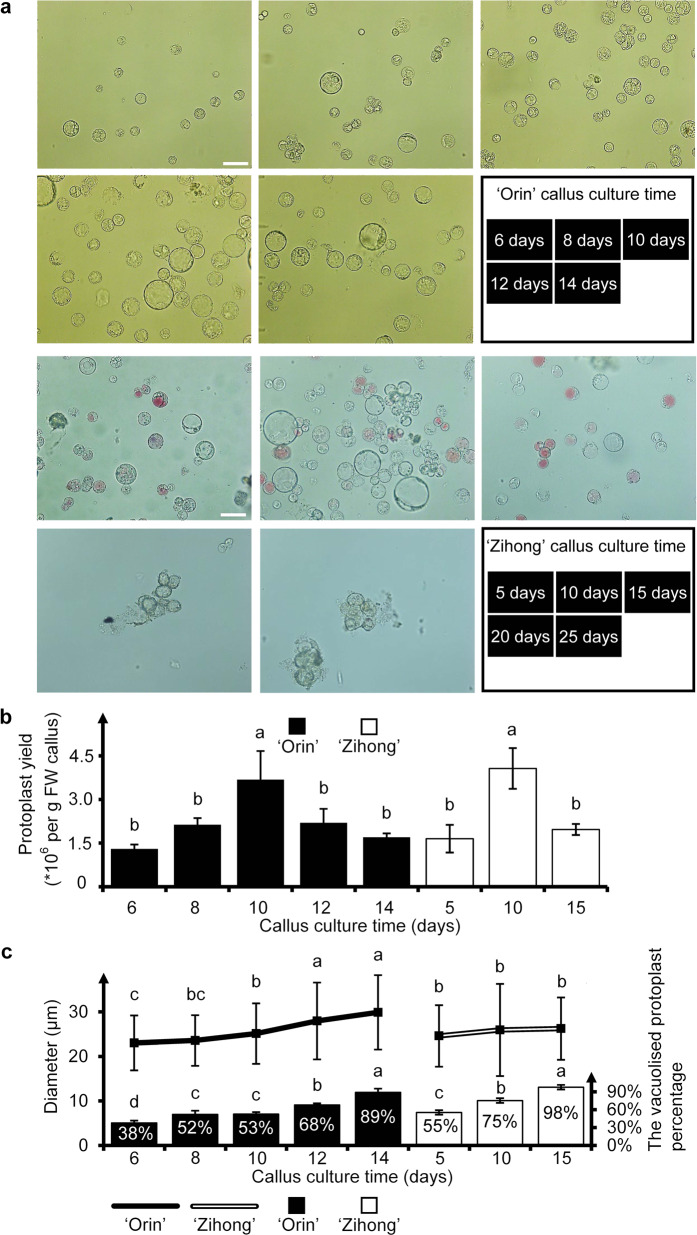
Protoplast cells isolated from apple callus cells cultured in MS medium for different durations. **a** ‘Orin’ (upper panel) and ‘Zihong’ (lower panel) apple protoplast cells under microscopy. The culture times of the apple callus cells used for protoplast isolation are shown to the right of the lower row in each panel. The scale bars represent 50 µm. **b** Protoplast yield of ‘Orin’ (filled columns) and ‘Zihong’ callus cells (open columns). FW fresh weight. **c** Average diameters (polyline) and percentage ratios of vacuolated cells (columns) in ‘Orin’ (filled symbols) and ‘Zihong’ (open symbols) protoplast cells (*n* ≥ 100). One-way ANOVA and multiple comparisons via Fisher’s LSD test were performed (*p* < 0.05). Different characters at the top of the columns indicate significant differences. Three independent experiments were conducted, and one representative result is shown

Intact apple protoplast cells that were spherical in shape and did not exhibit bursting or clustering were isolated from ‘Orin’ and ‘Zihong’ apple callus cells cultured in MS medium for different durations (Fig. 1a). The highest protoplast amounts were obtained from both ‘Orin’ and ‘Zihong’ apple callus cells cultured in MS medium for 10 days, yielding 3.68 × 10^6^ and 4.07 × 10^6^ protoplasts per gram of fresh weight callus (per g FW callus), respectively (Fig. 1b). Although apple callus cells cultured in MS medium for 6 days yielded only 1.3 × 10^6^ protoplasts per g FW, this condition yielded the strongest target protein expression (Supplementary File 1: Fig. S3a) and was therefore used for protoplast transfection in our research.

### Pro-BIUTNT is a potent promoter that enables the efficient expression of target genes in apple protoplast cells

**Fig. 2:**
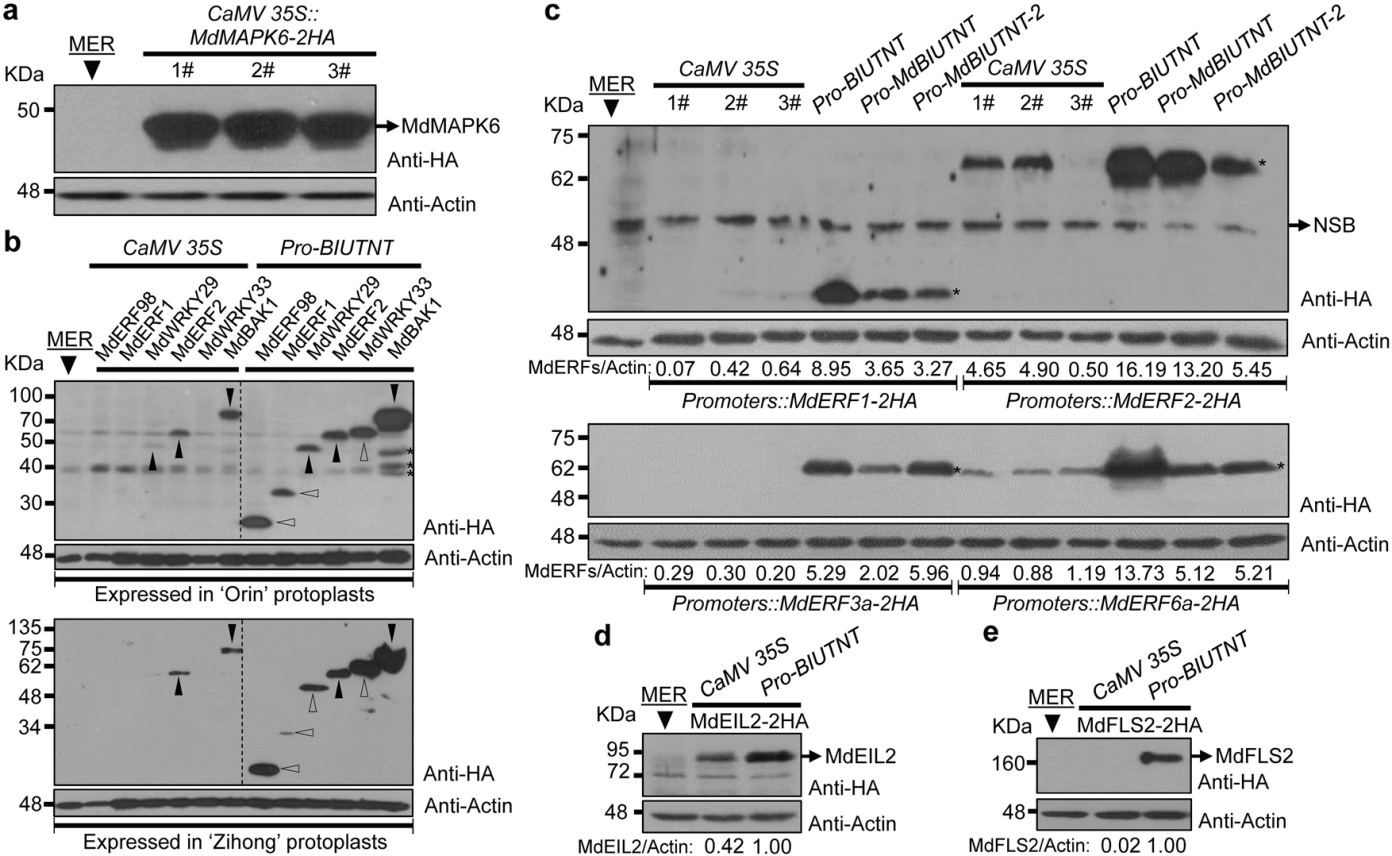
Stable and efficient expression of the tested genes in apple protoplast cells driven by *AtUBQ10* (At4g05320) or *MdUBQ10* (MDP0000820500) promoters. **a** MdMAPK6 expression driven by *CaMV 35S*. The expression of three independent clones is shown. **b** The expression of the tested genes in apple protoplast cells driven by *CaMV 35S* and *Pro*-*BIUTNT*. Filled and open triangles indicate weaker or stronger signals and the absence or presence of signals driven by *CaMV 35S* and *Pro*-*BIUTNT*, respectively. The asterisks indicate three processed bands of MdBAK1. **c** Further comparison of the abilities of the *CaMV 35S* and *UBQ10* promoters to drive the expression of the tested genes in apple protoplast cells. Three independent clones of the expression vector using *CaMV 35S* were selected. Asterisks indicate the signals of the expressed proteins. NSB nonspecific band. *Pro*-*BIUTNT*, the 1307-base pair (bp) sequence upstream from the *AtUBQ10 ATG* translational start codon; *Pro-MdBIUTNT*, the 1539-bp sequence upstream from the *MdUBQ10 ATG* translational start codon; *Pro-MdBIUTNT-2*, the 2501-bp sequence upstream from the *MdUBQ10 ATG* translational start codon. **d** MdEIL2 expression driven by the *CaMV 35S* and *Pro*-*BIUTNT* promoters. **e** MdFLS2 expression driven by the *CaMV 35S* and *Pro*-*BIUTNT* promoters. The intensities of the expressed proteins relative to that of actin are shown in **c**–**e**. The signal intensity was calculated by ImageJ. Actin was used as the internal control. MER represents a control sample transfected with the expression vector in which the target genes were replaced with a meaningless random sequence

Ethylene response factors (ERFs) are a large family of transcription factors that are extensively involved in controlling fruit ripening, disease resistance and other physiological activities or traits. MdERF1 and MdERF2 are two ERF transcription factors involved in controlling fruit ripening^25^. In our apple protoplast transient expression system driven by the *cauliflower mosaic virus* (*CaMV*) *35S* promoter, MdERF1 expression was not detectable, and a weak MdERF2 signal was detected by western blot. However, a clear and specific MdERF1 signal and a strong MdERF2 signal were detected when the promoter of *Arabidopsis ubiquitin-ten* (*Pro-BIUTNT*) was utilized (Fig. 2b), which is the 1307 nucleotide sequence upstream of the *ATG* translational start codon of the *Arabidopsis ubiquitin-10* (At4g05320) gene. Further studies revealed that two of the three independent MdERF2 clones under the control of *CaMV 35S* generated a dramatically weaker signal than that generated using *Pro-BIUTNT* (Fig. 2c). Only trace amounts of MdERF1 were detected in two MdERF1 clones under the control of *CaMV 35S* compared with the significantly stronger and more specific signal of the gene driven by *Pro-BIUTNT* (Fig. 2c).

MdERF3 and MdERF6 are two ERF transcription factors that are responsive to pathogen infection^26^. Homologs of MdERF3 and MdERF6, MdERF3a and MdERF6a were cloned and transfected into apple protoplast cells. Strong MdERF3a and MdERF6a signals were detected when the genes were driven by *Pro-BIUTNT*, but little or no signal was detected when the genes were driven by *CaMV 35S* (Fig. 2c).

ERF98 (At3g23230) is an ERF transcription factor that is responsive to treatment with flg22, a conserved 22-amino acid peptide from eubacterial flagellin that induces immune responses in plants^27^, and MdERF98 is the ortholog of ERF98 in the apple genome. No expression was found when driven by *CaMV 35S*, but a strong signal was detected for MdERF98 driven by *Pro-BIUTNT* (Fig. 2b). Analysis of a different *CaMV 35S*::*MdERF98* clone also revealed that the gene under the control of *CaMV 35S* was expressed at lower levels in protoplast cells than the gene driven by the *Pro-BIUTNT* promoter (Supplementary File 1: Fig. S1). This result suggests that *CaMV 35S* was not suitable for driving the expression of the target genes in apple protoplast cells and was consistent with the expression of three independent MdERF2 and MdERF1 clones driven by the *CaMV 35S* promoter in further assays.

MdEIL2 is the ortholog of EIN3 and regulates the expression of *MdPG1*^28^. A strong and clear MdEIL2 signal was achieved using *Pro-BIUTNT*, in contrast to the weak signal achieved with *CaMV 35S* (Fig. 2d).

WRKY33 and WRKY29 are involved in regulating ethylene and phytoalexin biosynthesis and pathogen-associated molecular pattern (PAMP)-triggered immune responses, respectively^29,30^. The orthologs of the two genes in apple, MdWRKY33 and MdWRKY29 (Table S1), were cloned and transfected into apple protoplast cells. MdWKY33 was hardly expressed under the control of *CaMV 35S*; however, a strong signal was obtained when it was driven by *Pro-BIUTNT* (Fig. 2b, Supplementary File 1: Fig. S3a, b). To further explore and compare the capabilities of *Pro-BIUTNT* and *CaMV 35S* to drive the expression of target genes in apple protoplast cells, the C-terminal epitope HA tag of MdWRKY33 was replaced with GFP. Strong MdWRKY33-GFP expression was detected when the gene was driven by *Pro-BIUTNT*, while no expression was found under the control of *CaMV 35S* (Supplementary File 1: Fig. S4a). *CaMV 35S* has numerous variants, and the variant we herein named *CaMV 35S-2* was subcloned from the pBI121 binary vector^31^ and analyzed for its ability to drive the expression of the target genes. Both of the *CaMV 35S* promoters contained the core sequence of the *CaMV 35S* promoter (Supplementary File 1: Sequences 1 and 2), and their abilities to drive the expression of target genes in apple have been proven^11^ (Fig. 4f). This variant of *CaMV 35S* resulted in weak MdERF98 expression and no MdWRKY33 expression (Supplementary File 1: Fig. S4b, c).

The above results suggested that *Pro-BIUTNT* is a strong promoter that enables the stable expression of target genes in apple protoplast cells.

**Fig. 3:**
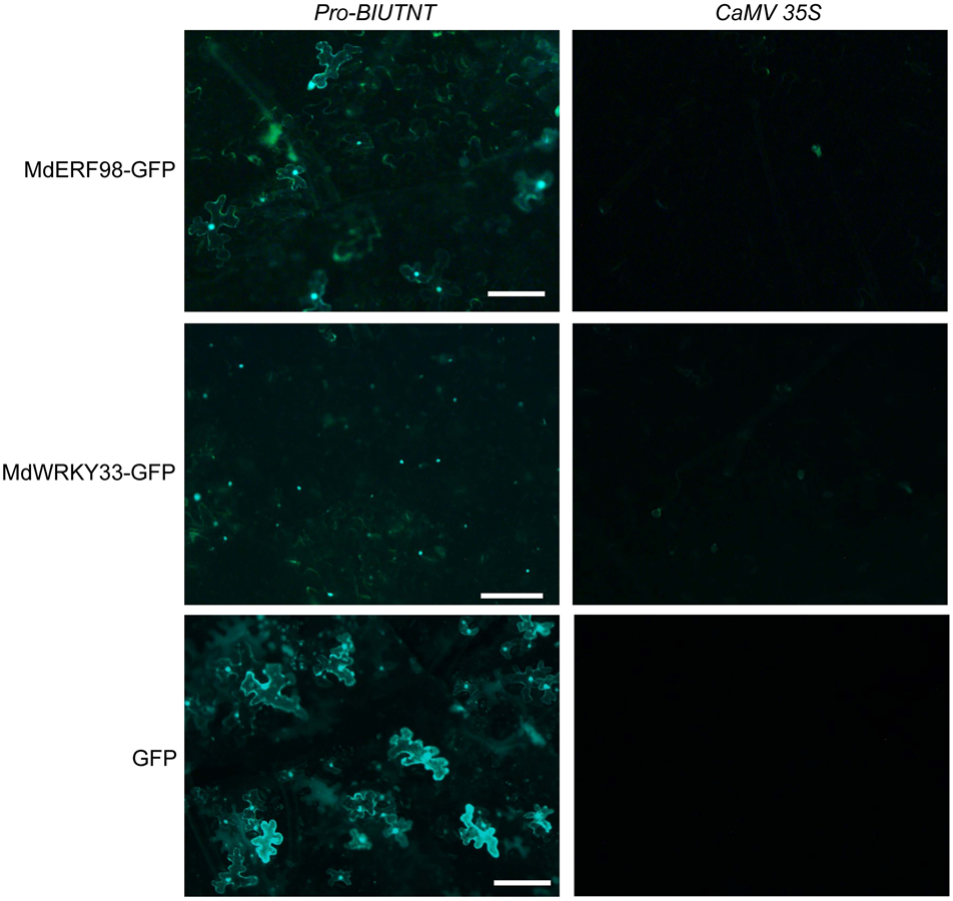
GFP and GFP-tagged proteins are strongly expressed in tobacco leaves under the control of the *AtUBQ10* (At4g05320) promoter. *Agrobacterium* harboring the target gene was infiltrated into the lower epidermal cells of the leaves of 5-week-old *Nicotiana benthamiana* plants grown under a photoperiod of 16-h light/8-h dark at 28 °C. After 3 days, the leaves were detached for GFP signal analysis under a microscope (Olympus, BX53F). *Pro*-*BIUTNT*, the 1307-base pair (bp) sequence upstream from the *AtUBQ10 ATG* translational start codon. Scale bar = 100 µm

Previous research showed that the *AtUBQ10* promoter was more stable and persistent than *CaMV 35S* in *Arabidopsis* and tobacco^32^, and the target gene was expressed at higher levels under the control of the *AtUBQ10* promoter in both dicot (*Arabidopsis*) and monocot (*rice*) plants^33^. Stronger WRKY33-GFP, MdERF98-GFP, and GFP expression in tobacco leaf epidermal cells was observed when they were driven by *Pro*-*BIUTNT* than when they were driven by *CaMV 35S* (Fig. 3), which was consistent with the stronger activity of the *AtUBQ10* promoter in apple protoplast cells (Fig. 2). Gene silencing contributes to the low expression of target genes driven by *CaMV 35S* in transgenic plants^33^. In apple, the expression level of the scab resistance gene *HcrVf2* under the control of *CaMV 35S* was 100-fold lower than that driven by the native promoter^12^. Whether gene silencing contributes to the low expression under the control of *CaMV 35S* in apple plants needs to be investigated further. However, the strong activity of *Pro-BIUTNT* and the contrasting low activity of *CaMV 35S* may be determined predominantly by the differences in the genetic backgrounds of apple and other plants, such as *Arabidopsis* and cotton^16,17,34^. *CaMV 35S* and *Pro-BIUTNT* showed similar activity levels in *Arabidopsis*, and while their activities were significantly reduced in apple protoplast cells, the activity of *Pro-BIUTNT* was ~5- to 10-fold higher than that of *CaMV 35S* (Supplementary File 1: Figs. S6, S2b). Another finding supporting our conclusion is that the promoter of *MdUBQ10* (MDP0000820500), the ortholog of *AtUBQ10* in the apple genome, drove the expression of target genes in apple protoplast cells significantly better than *CaMV 35S* (Fig. 2c). Notably, the *MdUBQ10* promoter sequence was not highly similar to that of *Pro-BIUTNT* (Supplementary File 1: Table S2).

Our research demonstrated that the *Pro-BIUTNT* and *MdUBQ10* promoter enable the efficient expression of target genes in apple protoplast cells.

### AtMKK7ac overexpression activates MAPK signaling in apple

MAPK signaling is an ancient and conserved signaling pathway in eukaryotes^35^ that helps to control plant growth, development, and disease resistance^30,35^. However, its upstream activators have not been identified in apple.

**Fig. 4:**
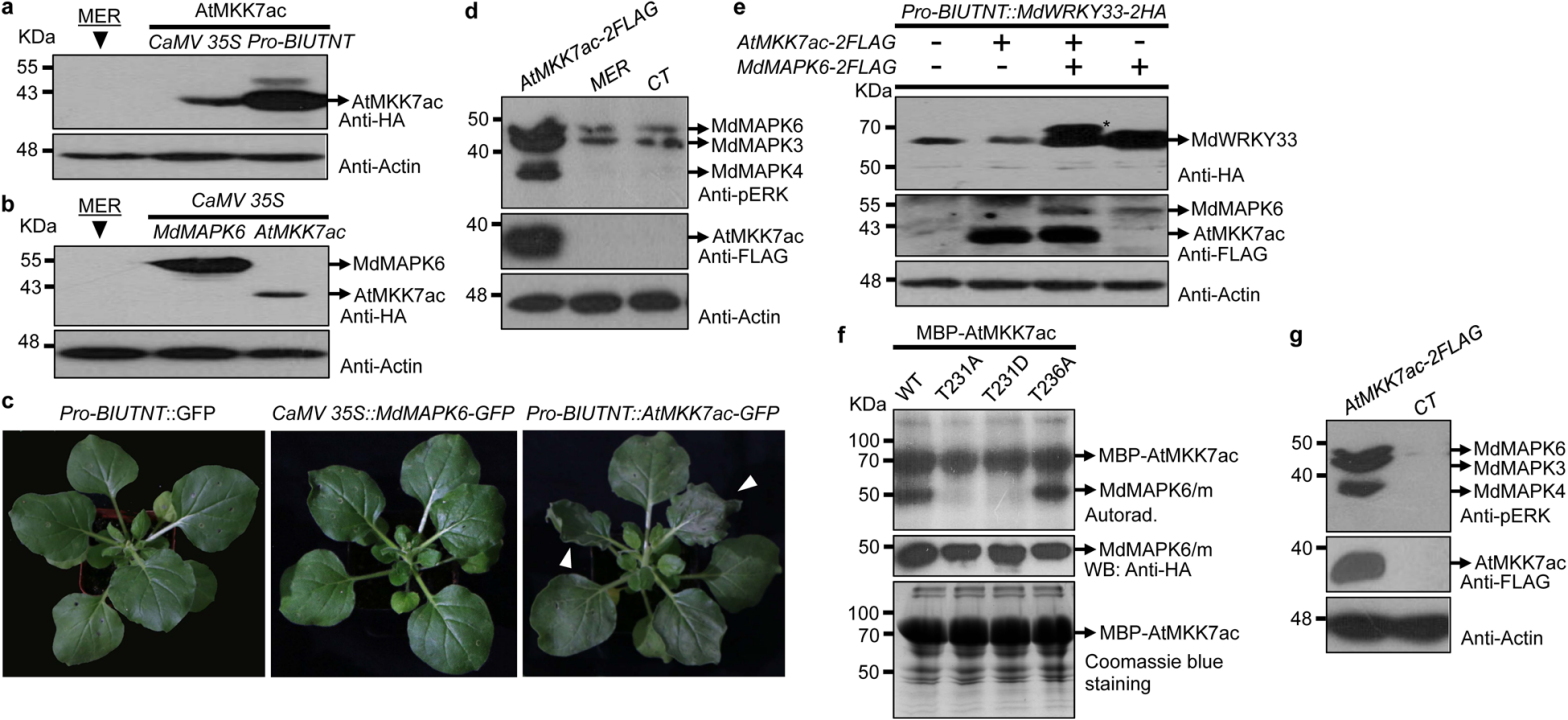
Overexpression of AtMKK7 in the active form (AtMKK7ac) activates MAPK signaling in apple protoplast cells. **a** Comparison of AtMKK7ac expression driven by *CaMV 35S* and *Pro*-*BIUTNT*. **b** Comparison of AtMKK7ac and MdMAPK6 expression driven by *CaMV 35S*. **c** AtMKK7ac overexpression induced hypersensitive responses in tobacco leaves. The white arrows denote the phenotype of hypersensitive reactions. *Agrobacterium tumefaciens* LBA 4404 carrying GFP, MdMAPK6-GFP, or AtMKK7ac-GFP was infiltrated into the leaves of 30-day-old tobacco seedlings, and the phenotypes were characterized 2 days later. **d** AtMKK7ac overexpression activates MAPK cascades in apple protoplast cells. AtMKK7ac was expressed in apple protoplast cells, and MAPK phosphorylation was detected by an anti-pERK antibody. CT untransformed protoplast cells. MER represents the control sample (**a**, **b**, **d**). **e** AtMKK7ac overexpression activates MdWRKY33 posttranslational modification, potentially phosphorylation, in apple protoplast cells. MdWRKY33-HA was expressed or coexpressed with AtMKK7ac-FLAG in protoplast cells generated from wild-type apple callus cells or from MdMAPK6-FLAG-overexpressing apple callus cells. MdWRKY33-HA was detected by western blot using an anti-HA antibody. **f** AtMKK7ac phosphorylates MdMAPK6 or activates MdMAPK6 autophosphorylation, which is mediated by Thr-231. An in vitro kinase assay was conducted as described in the Materials and methods section. The mutation of T231A/D but not T236A in MdMAPK6 blocked MdMAPK6 phosphorylation. Upper: phosphorylation statuses of MBP-AtMKK7ac and MdMAPK6 as determined by autoradiography; middle: expression of MdMAPK6 and its mutants in apple protoplast cells; lower: Coomassie blue staining of MBP-AtMKK7ac in the reaction samples. **g** AtMKKac overexpression activates MdMAPK3/6/4 phosphorylation in apple callus cells. Apple calli transformed with *Agrobacterium* carrying AtMKK7ac were placed on callus maintenance medium for 2 days. Apple calli transformed with *Agrobacterium* carrying an empty vector were used as the control (CT). MAPK phosphorylation was detected as described in **d**

AtMKK7 (At1g18350) is thought to be involved in regulating both basal and systemic acquired resistance in plants^36^. The constitutive expression of AtMKK7 in its active form (AtMKK7ac: AtMKK7 with S193ES199D mutation) induces hypersensitive reactions in tobacco leaves (Fig. 4c). The in vivo detection of MdMAPK phosphorylation using an anti-pERK antibody showed that the constitutive expression of AtMKK7ac in apple protoplast cells markedly activated the phosphorylation of MdMAPK3/6/4, particularly MdMAPK4 (Fig. 4d). AtMKK7ac was then expressed in apple callus cells via *Agrobacterium*-mediated transient expression, and AtMKK7ac overexpression strongly activated MdMAPK phosphorylation (Fig. 4g). An in vitro kinase assay showed that MBP-AtMKK7ac could phosphorylate MdMAPK6, and Thr-231 was important for this process (Fig. 4f).

In this study, apple protoplasts were isolated from genetically transformed ‘Orin’ apple callus cells expressing MdMAPK6-FLAG driven by *CaMV 35S* and from wild-type ‘Orin’ apple callus cells. The coexpression of AtMKK7ac and MdWRKY33 induced a band shift of MdWRKY33 in apple protoplast cells overexpressing MdMAPK6-FLAG, which mimicked the band shift of Botrytis-induced kinase 1 (BIK1) observed in PAMP-triggered immune responses^37^, whereas a similar band shift was not observed in protoplast cells expressing only MdWRKY33. This result suggested that the expression of MdWRKY33 in apple protoplast cells was modified, potentially by phosphorylation, by upstream activators. The level of MdWRKY33 in the presence of MdMAPK6-FLAG was markedly higher than that obtained without MdMAPK6-FLAG overexpression (Fig. 4e), which suggests that the stability of MdWRKY33 is influenced by the status and degree of its phosphorylation upon activation by upstream factors.

The revelation that AtMKK7ac activates MdMAPK6 provides an effective method for activating MAPK signaling in apple cells and for elucidating the elements controlling MdMAPK6 activation in apple.

### Pro-BIUTNT enables the reconstitution of BAK1 and FLS2 interaction in apple protoplast cells

**Fig. 5:**
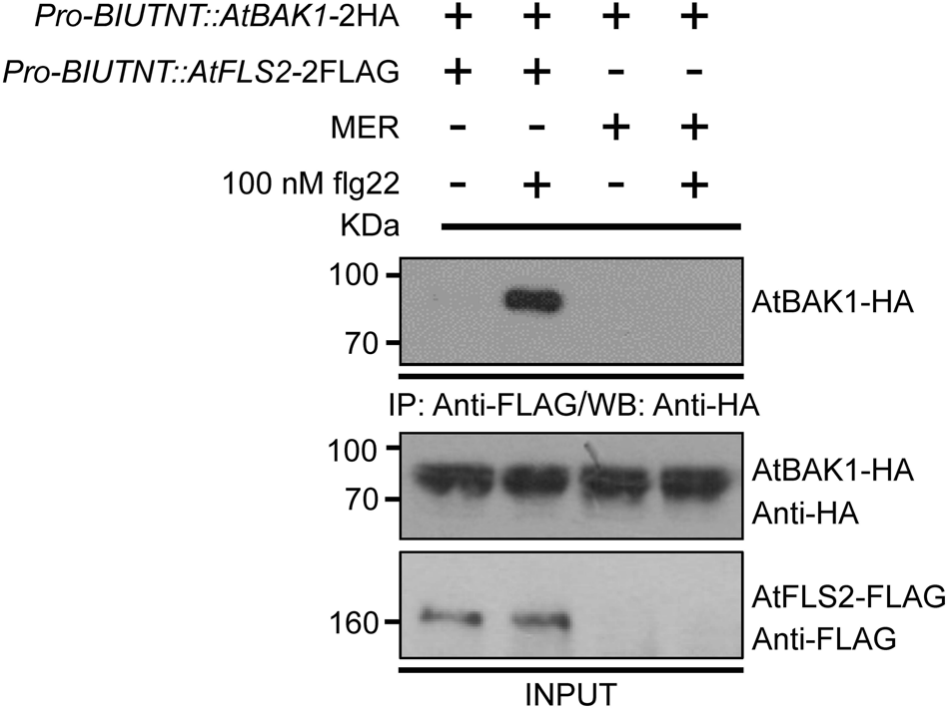
Reconstitution of the AtBAK1 and AtFLS2 interaction in apple protoplast cells. The interaction was detected by coimmunoprecipitation as described in the Materials and methods section. For the negative control, *Pro*-*BIUTNT*::*AtFLS2*-2FLAG was replaced by MER and cotransfected with *Pro*-*BIUTNT*::*AtBAK1*-2HA

The interaction of AtBAK1 and AtFLS2 is an important and fundamental signaling event in plant immune responses triggered by PAMPs^38^. Immune complex formation was observed in apple protoplast cells after flg22 elicitation. In this study, AtFLS2 with a C-terminal epitope tag FLAG (AtFLS2-FLAG) and AtBAK1 with an HA tag (AtBAK1-HA) were coexpressed in apple protoplast cells driven by *Pro-BIUTNT*. A coimmunoprecipitation assay was then performed to investigate the interaction between the two proteins in protoplasts. The AtBAK1-HA signal was found in the AtFLS2-FLAG complex immunoprecipitated from apple protoplast cells treated with flg22, whereas no signal was detected in AtFLS2-FLAG complexes immunoprecipitated from apple protoplasts that were not subjected to flg22 elicitation or from protoplasts expressing only MdBAK1-2HA (Fig. 5).

This study elucidated the paradigmatic signaling event in apple protoplast cells and provides a direct method for identifying novel signaling components of PAMP-triggered immune responses in highly developed perennial woody plants. This investigation also provides an accurate and convenient method for comparative studies of immune signaling in model and highly developed plants.

### Feedback regulation of AvrRpt2 by auxin and the RIN4-associated complex

AvrRpt2, a type III effector from *Pseudomonas syringae*, promotes pathogen virulence by enhancing auxin signaling and activates disease resistance by cleaving RIN4 in plants^39,40^. We previously showed that AvrRpt2 enhances auxin signaling by accelerating the turnover of the auxin signaling transcriptional repressor AXR2^40^.

**Fig. 6:**
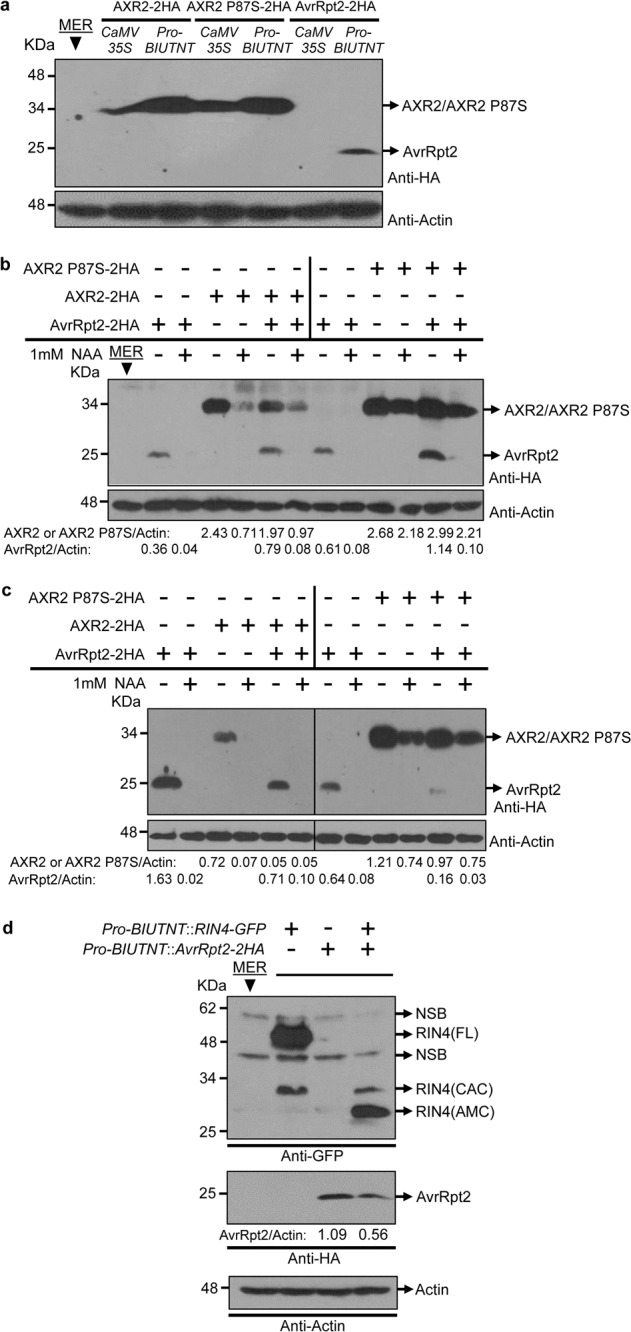
Negative and feedback regulation of AvrRpt2 by auxin signaling and the RIN4-associated complex. **a** AXR2, AXR2 P87S, and AvrRpt2 expression in apple protoplasts driven by *CaMV 35S* or *Pro-BIUTNT*. Negative regulation of AvrRpt2 by auxin signaling. AXR2 or AXR2 P87S was expressed or coexpressed with AvrRpt2 in apple protoplast cells (**b**) or tobacco leaves (**c**). The transfected protoplasts were incubated for 4 h and then treated with 1 mM NAA for another 2 h. AXR2 or AXR2 P87S was also expressed or coexpressed with AvrRpt2 in tobacco leaves for 3 days and then collected for treatment with 1 mM NAA for 6 h. The accumulation of proteins was detected by western blot using an anti-HA antibody. The signal intensities were calculated by ImageJ. The intensities of AXR2, AXR2 P87S, and AvrRpt2 relative to that of actin are shown beneath the actin signal. **d** Negative regulation of AvrRpt2 by the RIN4-associated complex. C-terminal epitope-tagged RIN4 with GFP (RIN4-GFP) and AvrRpt2 tagged with HA (AvrRpt2-HA) were coexpressed, driven by *Pro-BIUTNT*, in apple protoplast cells for 6 h. Protoplasts expressing only RIN4-GFP or AvrRpt2-HA were used as controls. Western blot analysis was conducted to detect the accumulation of the expressed proteins using anti-GFP (upper panel) or anti-HA antibodies (middle panel). Actin was used as the internal standard. The intensity of AvrRpt2-HA relative to that of actin (AvrRpt2/Actin) is shown. MER was used as the negative control. The experiment was repeated three times, and one representative result is shown. RIN4 (CAC) cell autologous cleavage-released band of RIN4, RIN4 (AMC) GFP-tagged C-terminus of RIN4 released by AvrRpt2-mediated cleavage, RIN4 (FL) full-length version of RIN4, NSB nonspecific band

Consistent with previous findings, AXR2 expression was significantly reduced in the presence of AvrRpt2 and 1-naphthalacetic acid (NAA), and the P87S mutation in AXR2 blocked NAA- and AvrRpt2-mediated degradation (Fig. 6b). Interestingly, the protein level of AvrRpt2 was markedly decreased to undetectable levels in the presence of 1 mM NAA (Fig. 6b). The same results were found in tobacco leaves when AvrRpt2 was expressed or coexpressed together with AXR2 or AXR2 P87S (Fig. 6c).

The degradation of AvrRpt2 was also identified in its interactions with RIN4. The functional processing of RIN4 by AvrRpt2 was confirmed by the disappearance of full-length RIN4-GFP [indicated as RIN4 (FL) in Fig. 6d] and the detection of the GFP-tagged RIN4 C-terminus [indicated as RIN4 (AMC) in Fig. 6d] upon the coexpression of RIN4-GFP with AvrRpt2-HA. We also repeatedly found that the AvrRpt2 level was decreased or even undetectable in the presence of RIN4 (Fig. 6d, Supplementary File 1: Fig. S5a, b).

These results suggest that the microbial effector is negatively regulated by auxin and the RIN4-associated complex in the plant. This phenomenon might represent two important disease resistance mechanisms in plant basal and effector-triggered resistance. The strengthened auxin signaling might provide feedback to remove the virulence source inside cells and thereby enhance plant disease resistance. In AvrRpt2-activated disease resistance mediated by the nucleotide-binding domain leucine-rich repeat (NLR) protein RPS2, AvrRpt2 might be negatively controlled to prevent the excessive induction of immune responses triggered by the causal agent itself. The detailed mechanisms by which auxin and the RIN4 complex control the microbial effector in plant cells will be elucidated in the future.

## Materials and methods

### Plasmid construction

*Pro-BIUTNT*, an abbreviation for ‘promoter of *ubiquitin-ten*’, the 1307-base pair (bp) sequence upstream from the translational start codon *ATG* of *Arabidopsis ubiquitin-10* (At4g05320), was amplified from *Arabidopsis* genomic DNA.

pHBT-*CaMV 35S*::*AvrRpm1*-2HA was used as the backbone for the construction of the protoplast transient expression vectors used in this study. The vector driven by *CaMV 35* *S* was constructed by directly replacing *AvrRpm1* with the coding sequence of the target gene. The vector driven by the *Pro-BIUTNT* or *MdUBQ10* (MDP0000820500) promoter was constructed by substituting *Pro-BIUTNT* or nucleotide sequences of different lengths upstream from the *ATG* translational start codon of *MdUBQ10* for *CaMV 35S*.

### Protoplast isolation, transfection, and western blot analysis

Apple protoplast cells were isolated from ‘Orin’ and ‘Zihong’ apple callus cells that had been cultured in MS medium for different durations according to the method described by Gao et al.^34^. One gram of apple callus cells was dispersed evenly in 10 mL of enzyme digestion solution consisting of 1.5% cellulase, 0.4% macerozyme, and 0.05% pectinase dissolved in 20 mM MES buffer containing 20 mM KCl, 10 mM CaCl_2_, 2% sucrose, and 0.4 M mannitol (pH adjusted to 5.7). After vacuum infiltration for 30 min, the digestion solution was maintained undisturbed at room temperature for 8 h. The solution was then passed through a nylon mesh with a diameter of 100 µm and collected in a 50 mL Eppendorf tube, to which an equal volume of W5 solution was added. The nylon mesh was washed twice with W5 solution, and the solution was collected and added to the protoplast cell suspension. After the suspension was centrifuged at 650 × *g* for 2 min, the protoplast cells were collected, resuspended in 15 mL of W5 solution, and maintained on ice for 30 min. The supernatant was then discarded, and the protoplast cells were suspended in 1 mL of MMG solution and later used for protoplast cell number calculations and plasmid DNA transfection.

Protoplasts were transfected as described by He et al.^16^. Protein expression in protoplast cells was detected by western blot using anti-HA, anti-FLAG, or anti-GFP antibodies.

### Luciferase activity assay

*AvrRpm1* in pHBT-*CaMV 35S*::*AvrRpm1*-2HA was replaced with a luciferase (Luc) coding sequence to generate pHBT-*CaMV 35S*::*Luc*. *CaMV 35S* was then replaced to generate luciferase vectors under the control of the promoters investigated in the research. The luciferase activity in apple or *Arabidopsis* protoplast cells was measured according to He et al.^16^.

### Co-immunoprecipitation (Co-IP)

Driven by *Pro-BIUTNT*, AtBAK1-HA and AtFLS2-FLAG were expressed in apple protoplast cells for 6 h. After treatment with 100 nM flg22 or the equivalent amount of solvent for an additional 10 min, the protoplast cells were harvested. Co-IP was conducted using anti-FLAG beads according to Lu et al.^37^.

Cells expressing AtBAK1 that were treated or not treated with only flg22 were used as controls.

### In vitro kinase assay

MdMAPK6 (MDP0000340624) or its mutant MdMAPK6 T231A, MdMAPK6 T231D, or MdMAPK6 T236A was cloned into a pBI121 binary vector under the control of *CaMV 35S* with a C-terminal FLAG epitope tag and transformed into apple callus cells according to He et al.^11^.

The recombinant protein MBP-AtMKK7ac was induced and purified from *E. coli* BL21.

In the kinase activity assay, MdMAPK6 or one of its mutants was purified from one gram of transgenic apple callus cells and mixed with two micrograms of MBP-AtMKK7ac in 25 µL of kinase reaction buffer (20 mM Tris-HCl [pH 7.5], 5 mM EDTA [pH 8.0], 1 mM DTT, 20 mM MgCl_2_, 0.1 mM ATP and 5 µCi [γ-^32^P] ATP). After reacting for 2 h at 23 °C, the proteins were denatured and separated by SDS-PAGE. Phosphorylation was detected by autoradiography.

## Supplementary information

Supplementary file 2

Supplementary file 1

## Data Availability

All data supporting the findings of this study are available within the paper or within its supplementary information published online. The materials used in this study are available from the corresponding author upon reasonable request.

## References

[1] Yao JL, Dong YH, Morris BAM (2001). Parthenocarpic apple fruit production conferred by transposon insertion mutations in a MADS-box transcription factor. Proc. Natl Acad. Sci. USA.

[2] Ireland HS (2013). Apple SEPALLATA1/2-like genes control fruit flesh development and ripening. Plant J..

[3] Zhou K (2019). MdUGT88F1-mediated phloridzin biosynthesis regulates apple development and *Valsa* canker resistance. Plant Physiol..

[4] Chagné D (2013). An ancient duplication of apple MYB transcription factors is responsible for novel red fruit-flesh phenotypes. Plant Physiol..

[5] Li YY (2012). MdCOP1 ubiquitin E3 ligases interact with MdMYB1 to regulate light-induced anthocyanin biosynthesis and red fruit coloration in apple. Plant Physiol..

[6] Hu DG (2016). MdMYB1 regulates anthocyanin and malate accumulation by directly facilitating their transport into vacuoles in apples. Plant Physiol..

[7] Zhu L (2021). MdERDL6-mediated glucose efflux to the cytosol promotes sugar accumulation in the vacuole through up-regulating TSTs in apple and tomato. Proc. Natl Acad. Sci. USA.

[8] Wang N (2017). MYB12 and MYB22 play essential roles in proanthocyanidin and flavonol synthesis in red-fleshed apple (*Malus sieversii* f. *niedzwetzkyana*). Plant J..

[9] Li Y (2013). Interactions of apple and the *Alternaria alternate* apple pathotype. Crit. Rev. Plant Sci..

[10] Meng D (2018). Sorbitol modulates resistance to *Alternaria alternata* by regulating the expression of an *NLR* resistance gene in apple. Plant Cell.

[11] He X (2018). Activation of disease resistance against *Botryosphaeria dothidea* by downregulating the expression of *MdSYP121* in apple. Hort. Res.

[12] Gessler C, Pertot H (2012). Vf scab resistance of *Malus*. Trees.

[13] Li C (2020). A sugar transporter takes up both hexose and sucrose for sorbitol-modulated in vitro pollen tube growth in apple. Plant Cell.

[14] Meng D (2014). Apple MdABCF assists in the transportation of S-RNase into pollen tubes. Plant J..

[15] Spolaore S, Trainotti L, Casadoro G (2001). A simple protocol for transient gene expression in ripe fleshy fruit mediated by, *Agrobacterium*. J. Exp. Bot..

[16] He, P., Shan, L. & Sheen, J. in Methods in Molecular Biology, Vol. 354 (ed Ronald, P. C.) Ch. 1 (Humana, 2010).

[17] Ma X (2020). Ligand-induced monoubiquitination of BIK1 regulates plant immunity. Nature.

[18] Zhang XC, Millet YA, Cheng Z, Bush J, Ausubel FM (2015). Jasmonate signalling in *Arabidopsis* involves SGT1b–HSP70–HSP90 chaperone complexes. Nat. Plants.

[19] Fu L (2021). The TOR–EIN2 axis mediates nuclear signalling to modulate plant growth. Nature.

[20] Zhou Y (2018). Telobox motifs recruit CLF/SWN-PRC2 for H3K27me3 deposition via TRB factors in. Arabidopsis. Nat. Genet..

[21] Zhang N (2018). Engineering artificial microRNAs for multiplex gene silencing and simplified transgenic screen. Plant Physiol..

[22] Wu S (2020). Establishment of a PEG-mediated protoplast transformation system based on DNA and CRISPR/Cas9 ribonucleoprotein complexes for banana. BMC Plant Biol..

[23] Osakabe Y (2018). CRISPR–Cas9-mediated genome editing in apple and grapevine. Nat. Protoc..

[24] Marion J (2008). Systematic analysis of protein subcellular localization and interaction using high-throughput transient transformation of *Arabidopsis* seedlings. Plant J..

[25] Li T (2017). The jasmonate-activated transcription factor MdMYC2 regulates ETHYLENE RESPONSE FACTOR and ethylene biosynthetic genes to promote ethylene biosynthesis during apple fruit ripening. Plant Cell.

[26] Akagi A, Dandekar AM, Stotz HU (2011). A resistance of *Malus domestica* fruit to *Botrytis cinerea* depends on endogenous ethylene biosynthesis. Phytopath.

[27] Wu S (2011). Bacterial effector HopF2 suppresses *Arabidopsis* innate immunity at the plasma membrane. Mol. Plant-Microbe Interact..

[28] Tacken E (2010). The role of ethylene and cold temperature in the regulation of the apple polygalacturonase1 gene and fruit softening. Plant Physiol..

[29] Zhou J (2020). Differential phosphorylation of the transcription factor WRKY33 by the protein kinases CPK5/CPK6 and MPK3/MPK6 cooperatively regulates camalexin biosynthesis in *Arabidopsis*. Plant Cell.

[30] Asai T (2002). MAP kinase signalling cascade in *Arabidopsis* innate immunity. Nature.

[31] Li J, An B, Zhang X (2012). Identification and promoter analysis of some important storage protein genes from wheat (*Triticum aestivum* L.). Plant Omics J..

[32] Grefen C (2010). A ubiquitin-10 promoter-based vector set for fluorescent protein tagging facilitates temporal stability and native protein distribution in transient and stable expression studies. Plant J..

[33] Behera S (2015). Analyses of Ca^2+^ dynamics using a ubiquitin-10 promoter-driven Yellow Cameleon 3.6 indicator reveal reliable transgene expression and differences in cytoplasmic Ca^2+^ responses in *Arabidopsis* and rice (*Oryza sativa*) roots. N. Phytol..

[34] Gao X (2011). Silencing GhNDR1 and GhMKK2 compromises cotton resistance to *Verticillium* wilt. Plant J..

[35] Zhu Q (2019). A MAPK cascade downstream of IDA-HAE/HSL2 ligand-receptor pair in lateral root emergence. Nat. Plants.

[36] Zhang X (2007). Overexpression of *Arabidopsis* MAP kinase kinase 7 leads to activation of plant basal and systemic acquired resistance. Plant J..

[37] Lu D (2010). A receptor-like cytoplasmic kinase, BIK1, associates with a flagellin receptor complex to initiate plant innate immunity. Proc. Natl Acad. Sci. USA.

[38] de Oliveira MV (2016). Specific control of *Arabidopsis* BAK1/SERK4-regulated cell death by protein glycosylation. Nat. Plants.

[39] Djami-Tchatchou AT (2020). Dual role of auxin in regulating plant defense and bacterial virulence gene expression during *Pseudomonas syringae* PtoDC3000 pathogenesis. Mol. Plant-Microbe Interact..

[40] Cui F (2013). The *Pseudomonas syringae* type III effector AvrRpt2 promotes pathogen virulence via stimulating *Arabidopsis* auxin/indole acetic acid protein turnover. Plant Physiol..

