# Supplementary material for: Development of potent promoters that drive the efficient expression of genes in apple protoplasts
Source: Hortic Res. 2021 Oct 1;8:211. doi: 10.1038/s41438-021-00646-4 (PMC8484340; doi:10.1038/s41438-021-00646-4)
Supplement: Supplementary file 1 — Supplementary file 2 [file 41438_2021_646_MOESM1_ESM.docx]

**Supplementary file 2**

Table S1 Primers used in this study.

| **Primer name** | **Primer sequence (5’ - 3’)** | **Description** |
| --- | --- | --- |
| **Primers for the cloning of ubiquitin promoters used in the western blot analysis of protein expression** | | |
| Pro-BIUTNT-F | AA*CTGCAG* GTCAACGGATCAGGATATTCTTGTTTAAG | 1307 bp |
| Pro-BIUTNT-R | TCC*CCCGGG* CTGTTAATCAGAAAAACTCAG |  |
| Pro-BIUTNT-R | CG*GGATCC* CTGTTAATCAGAAAAACTCAG |  |
| Pro-MdBIUTNT-F | AA*CTGCAG* CCTTGAAAACAAATTATTTTTTCGG | 1539 bp |
| Pro-MdBIUTNT-2-F | AA*CTGCAG* GGATTTATGGCTGTGGATCGCCC | 2501 bp |
| Pro-MdBIUTNT-R | TCC*CCCGGG* GGTTTCAGAAATTGGGGATTTG |  |
| Pro-MdBIUTNT-R | CG*GGATCC* GGTTTCAGAAATTGGGGATTTG |  |
| Pro-MdRP-1-F | AA*CTGCAG* CCCACAACATCTCCATGGCTAAGTTGAG | 1972 bp |
| Pro-MdRP-2-F | AA*CTGCAG* CTTGTGTAGGTTGATGAATCTCTTAT | 3050 bp |
| Pro-MdRP-R | TCC*CCCGGG* CTGGAGCACACTTTGAAGCTGACA |  |
| Pro-MdRC-1-F | AA*CTGCAG* AGCCTTAGTATTATTATGACTGACA | 2025 bp |
| Pro-MdRC-2-F | AA*CTGCAG* TAACTTGAGACCAAGATAGCCACT | 2542 bp |
| Pro-MdRC-R | TCC*CCCGGG* GATCTCTCGCGGATCGGACGGTT |  |
| Pro-MdBIUTNT-339-F | AA*CTGCAG* AGCAAGGAAAGGGGCAACAAAAATC | 339 bp |
| Pro-MdBIUTNT-539-F | AA*CTGCAG* TCCGTCGATCTTCTTCAATTTATC | 539 bp |
| Pro-MdBIUTNT-739-F | AA*CTGCAG* TATTTTGAAAACAATTTTAAATC | 739 bp |
| Pro-MdBIUTNT-939-F | AA*CTGCAG* GGATGGCACGCCCGAACTTGATAC | 939 bp |
| Pro-MdBIUTNT-1139-F | AA*CTGCAG* AAATTTCTCCTTGGCGAAAATCG | 1139 bp |
| Pro-MdBIUTNT-1339-F | AA*CTGCAG* AACTACACAAAATAAGAGGGTAC | 1339 bp |
| Pro-MdBIUTNT-R | CG*GGATCC* GGTTTCAGAAATTGGGGATTTG |  |
| **Primers for the cloning of ubiquitin promoters used in the luciferase-based analysis of protein expression** | | |
| Luciferase-F | CG*GGATCC*ATGGAAGACGCCAAAAACAT |  |
| Luciferase-R | TCC*CCCGGG* CAATTTGGACTTTCCGCCCTTC |  |
| Pro-BIUTNT-F | AA*CTGCAG* GTCAACGGATCAGGATATTCTTGTTTAAG | 1307 bp |
| Pro-BIUTNT-R | CATG*CCATGG*CTGTTAATCAGAAAAACTCAG |  |
| Pro-MdBIUTNT-339-F | AA*CTGCAG* AGCAAGGAAAGGGGCAACAAAAATC | 339 bp |
| Pro-MdBIUTNT-539-F | AA*CTGCAG* TCCGTCGATCTTCTTCAATTTATC | 539 bp |
| Pro-MdBIUTNT-739-F | AA*CTGCAG* TATTTTGAAAACAATTTTAAATC | 739 bp |
| Pro-MdBIUTNT-939-F | AA*CTGCAG* GGATGGCACGCCCGAACTTGATAC | 939 bp |
| Pro-MdBIUTNT-1139-F | AA*CTGCAG* AAATTTCTCCTTGGCGAAAATCG | 1139 bp |
| Pro-MdBIUTNT-1339-F | AA*CTGCAG* AACTACACAAAATAAGAGGGTAC | 1339 bp |
| Pro-MdBIUTNT-F | AA*CTGCAG* CCTTGAAAACAAATTATTTTTTCGG | 1539 bp |
| Pro-MdBIUTNT-2-F | AA*CTGCAG* GGATTTATGGCTGTGGATCGCCC | 2501 bp |
| Pro-MdBIUTNT-R | CATG*CCATGG*GGTTTCAGAAATTGGGGATTTG |  |
| **Primers for the cloning of genes used in the western blot analysis of protein expression** | | |
| MdERF1-F | CG*GGATCC* ATGTGTGGTGGTGCTATCA |  |
| MdERF1-R | GA*AGGCCT* ATACAGAAGCTGCCCTTGTTG |  |
| MdERF2-F | CG*GGATCC* ATGGAAGAACAGGCTCAGCA |  |
| MdERF2-R | GA*AGGCCT* GCTAACCAATAGTTGCTCGCCAA |  |
| MdERF3a-F | CG*GGATCC* ATGTGCTTACTGAAGGTGGC |  |
| MdERF3a-R | GA*AGGCCT* ACTGGATGAGGATGGATTG |  |
| MdERF6a-F | CATG*CCATGG*GCTTACTGAAGGTG |  |
| MdERF6a-R | GA*AGGCCT* ACCGGATGAGGATGGATT |  |
| MdERF98-F | CG*GGATCC* ATGGAGGGGAAGAGAGGAC |  |
| MdERF98-F | TCC*CCCGGG* ATGGAGGGGAAGAGAGGAC |  |
| MdERF98-R | GA*AGGCCT* CTGTGTTGGTTGCCCCTGCCTAT |  |
| MdBAK1-F | CATG*CC ATGG*ACCCAACACTGATGAC |  |
| MdBAK1-R | TCC*CCCGGG* TCTGGGACCGGACAACTC |  |
| MdFLS2-F | CATG*CC ATGG*TGTCTCAGAGATTAAG |  |
| MdFLS2-R | TCC*CCCGGG* TGTTTCCCTTTTCAGCTTCAG |  |
| MdWRKY29-R | CG*GGATCC* ATGGAGAACTGGGATTTGC |  |
| MdWRKY29-F | TCC*CCCGGG* GCAAGCACCGGTTACAGTATTAG |  |
| MdWRKY33-F | CG*GGATCC* ATGACTTCTTCCTTCACTCAC |  |
| MdWRKY33-R | TCC*CCCGGG* GARCTCAGAAAATCCATAACTTC |  |
| AtBAK1-F | CG*GGATCC* ATGGAACGAAGATTAATGATC |  |
| AtBAK1-R | GA*AGGCCT* TCTTGGACCCGAGGGGTATTCG |  |
| AtFLS2-F | TCC*CCCGGG* ATGAAGTTACTCTCAAAGACC |  |
| AtFLS2-R | TCC*CCCGGG* AACTTCTCGATCCTCGTT |  |
| MdMAPK6-F | CG*GGATCC* ATGGAGGGAGGAGGGCGAT |  |
| MdMAPK6-R | GA*AGGCCT* CTGTCGCTGGTACTCGGGGTTA |  |
| AXR2-F | CG*GGATCC* ATGATCGGCCAACTTATGAACC |  |
| AXR2-R | GA*AGGCCT* AGATCTGTTCTTGCAGTACTTC |  |
| AvrRpt2-F | CG*GGATCC*atgaaaattgctccagttgcc |  |
| AvrRpt2-R | GA*AGGCCT* GCGGTAGAGCATTGCGTGTGG |  |
| RIN4-F | CG*GGATCC* ATGGCACGTTCGAATGTACCAA |  |
| RIN4-R | TCC*CCCGGG* TTTTCCTCCAAAGCCAAAGCAG |  |
| GFP-F | GA*AGGCCT*GTGAGCAAGGGCGAGGAG |  |
| GFP-R | TCC*CCCGGG*CCGGGCGGCCGCTTTACTTG |  |
| AtMKK7-F | TCC*CCCGGG* ATGGCTCTTGTTCGTAAACG |  |
| AtMKK7-F | CG*GGATCC* ATGGCTCTTGTTCGTAAACG |  |
| AtMKK7-R | GA*AGGCCT* AAGACTTTCACGGAGAAAAGGGTG |  |
| **Primers for gene mutation** | | |
| AXR2 P87S-F | CAAGTGGTGGGATGGTCACCTGTGAGGAACTAC |  |
| AXR2 P87S-R | GTAGTTCCTCACAGGTGACCATCCCACCACTTG |  |
| MdMAPK6 T231-F | AGATGGTACCGTGCCCCAGAGCTACTG |  |
| MdMAPK6 T231A-R | GGTAACAACATACTCGGCCATAAAATCAGTCTC |  |
| MdMAPK6 T231D-R | GGTAACAACATACTCGTCCATAAAATCAGTCTC |  |
| MdMAPK6 T236A-F | CCAGAGCTACTGTTAAACTCTTCAG |  |
| MdMAPK6 T236A-R | GGCACGGTACCATCTGGCAACAACATACTCAGT |  |
| AtMKK7ac-F | TGCGCTTACATGAGCCCGGAGAGATTTGACTCTG |  |
| AtMKK7ac-S193E/S199D-R | AGTGCCGACGTAATCATTGCAGTAATCTAACTCTCGGGTAATGAT |  |
| **Primers for the cloning of promoters used for transfection into *Nicotiana benthamiana*** | | |
| Pro-BIUTNT-F | CG*GGATCC* GTCAACGGATCAGGATATTCTTGTTTAAG |  |
| Pro-BIUTNT-R | TCC*CCCGGG* CTGTTAATCAGAAAAACTCAG |  |
| **Primers for the cloning of genes used for transfection into *Nicotiana benthamiana*** | | |
| AtMKK7-F | TCC*CCCGGG* ATGGCTCTTGTTCGTAAACG |  |
| AtMKK7-R | GA*AGGCCT* AAGACTTTCACGGAGAAAAGGGTG |  |
| MdMAPK6-F | CG*GGATCC* ATGGAGGGAGGAGGGCGAT |  |
| MdMAPK6-R | GA*AGGCCT* CTGTCGCTGGTACTCGGGGTTA |  |
