# Supplementary material for: Development of potent promoters that drive the efficient expression of genes in apple protoplasts
Source: Hortic Res. 2021 Oct 1;8:211. doi: 10.1038/s41438-021-00646-4 (PMC8484340; doi:10.1038/s41438-021-00646-4)
Supplement: Supplementary file 2 — Supplementary file 1 [file 41438_2021_646_MOESM2_ESM.docx]

**Supplementary file 1**

Table S1 The tested genes and their orthologs in *Arabidopsis thaliana* or apple.

| Apple gene  (Gene ID) | *Arabidopsis* gene  (Gene ID) | Amino acid  sequence  identity | Coding sequence  identity |
| --- | --- | --- | --- |
| ***MdMAPK6***  (MDP0000340624) | *AtMAPK6*  (AT2G43790) | 85.96% | 73.11% |
| ***MdERF98***  (MDP0000312701) | *AtERF98*  (AT3G23230) | 55.06% | 56.33% |
| ***MdERF1***  (AB288347) | *AtERF72*  (AT3G16770) | 40.15% | 52.35% |
| ***MdERF2***  (AB288348) | *AtERF2*  (AT5G47220) | 32.28% | 37.35% |
| ***MdERF3a***  (MDP0000242979) | *AtERF110*  (AT5G50080) | 25.33% | 25.11% |
| ***MdERF6a***  (MDP0000235028) | *AtABR1*  (AT5G64750) | 32.74% | 42.68% |
| ***MdBAK1***  (MDP0000291093) | ***AtBAK1***  (AT4G33430) | 73.93% | 75.78% |
| ***MdFLS2***  (MDP0000254122) | ***AtFLS2***  (AT5G46330) | 53.65% | 58.89% |
| ***MdEIL2***  (GU732485.1) | *AtEIN3*  (AT3G20770) | 35.28% | 44.43% |
| ***MdWRKY33***  (MDP0000296025) | *AtWRKY33*  (AT2G38470) | 44.20% | 53.11% |
| ***MdWRKY29***  (MDP0000247896) | *AtWRKY29*  (AT4G23550) | 38.46% | 49.59% |
| *MdRIN4*  (MDP0000178481) | ***RIN4***  (At3g25070) | 20.58% | 41.68% |
| *MdAXR2*  (MDP0000124810) | ***AXR2***  (At3G23050) | 48.41% | 47.70% |
| *MdMKK7*  (MDP0000252992) | ***AtMKK7***  (At1G18350) | 60.63% | 64.66% |
| *Pseudomonas* gene  (Gene ID) |  |  |  |
| ***AvrRpt2***  (Z21715.2) | NA | NA | NA |

Note: The tested genes in our research are in bold. NA: not applicable

Table S2 The identities of *AtUBQ10* (At4g05320) and *MdUBQ10* (MDP0000820500) in the promoter region.

| **Nucleic acid base pairs** | **Identity (nucleotide)** |
| --- | --- |
| 339 | 33.33% |
| 539 | 37.17% |
| 739 | 36.94% |
| 939 | 37.12% |
| 1139 | 38.16% |
| 1307 | 38.44% |
| Full length  (1307 *vs* 1539) | 34.02% |

Note: Nucleotide sequences of different lengths upstream from the *ATG* translational start codon were compared. *Pro-BIUTNT* was compared with the same number of base pairs in the *MdUBQ10* promoter region and with *Pro-MdBIUTNT*, the 1539-bp sequence upstream from the *MdUBQ10* translational start codon


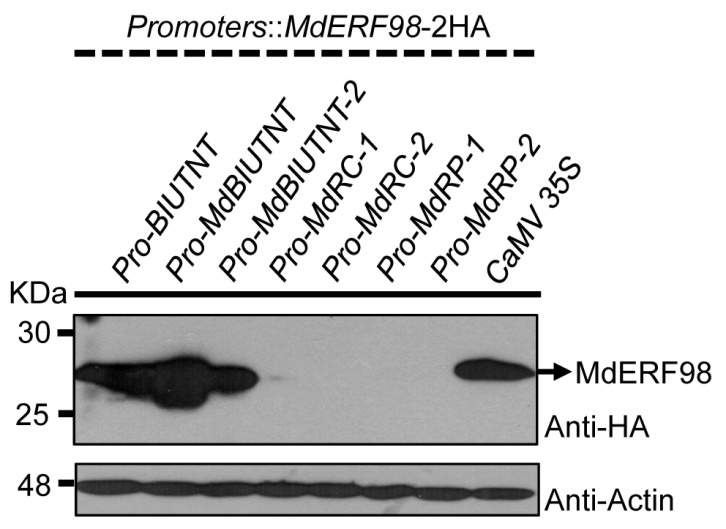


**Fig. S1 MdERF98 expression driven by different promoters.** *Pro-MdRP-1/2* represents the 1972/3050-bp sequence upstream from the *ATG* translational start codon of the apple *rubisco* small subunit gene (MDP0000316929). *Pro-MdRC-1/2* represents the 2025/2542-bp sequence upstream from the *ATG* translational start codon of the apple *rubisco activase* gene (MDP0000321244)


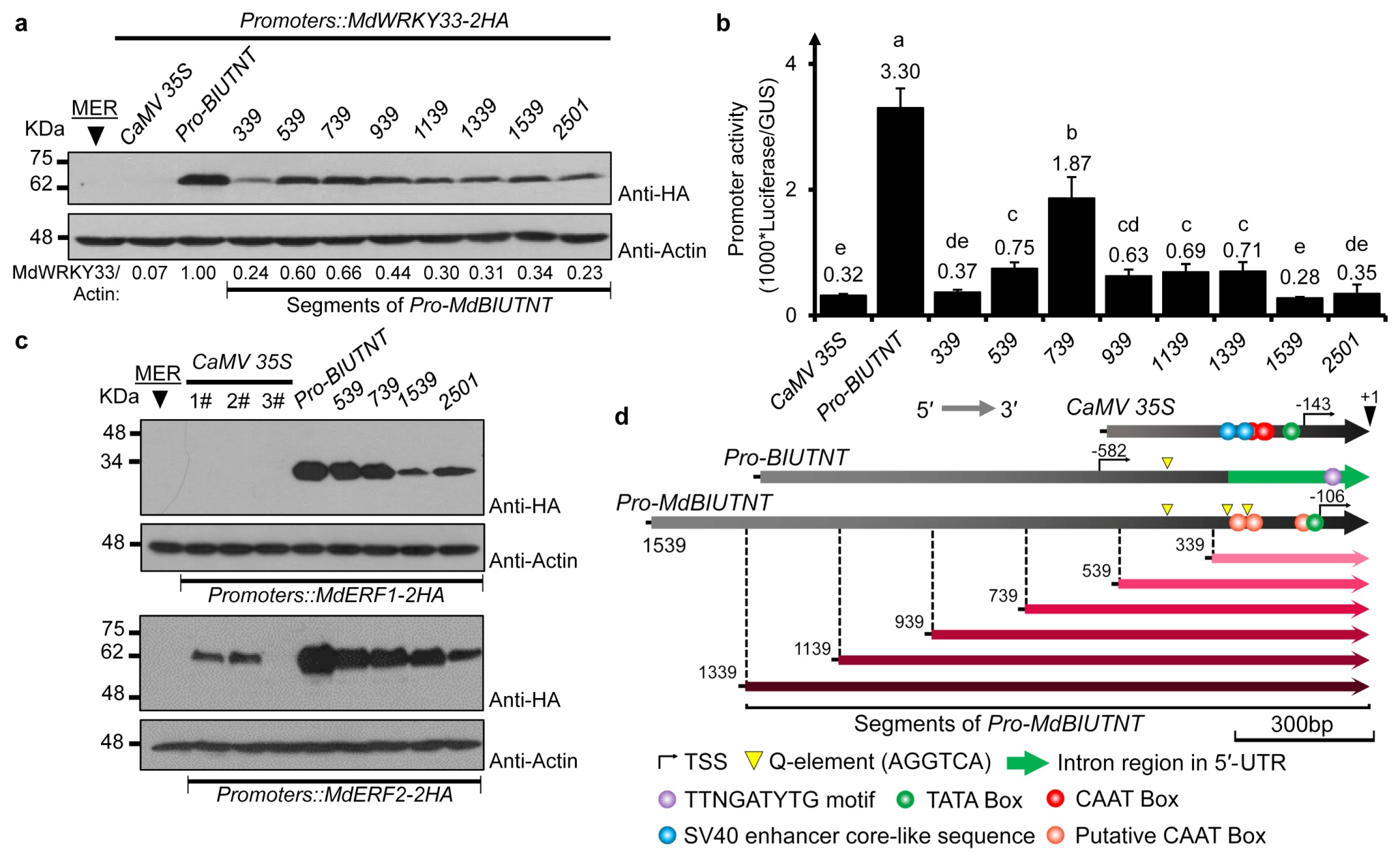


**Fig. S2 The sequences 539 and 739 bp upstream from the *ATG* translational start codon of *MdUBQ10* have the maximum ability to drive protein expression. a** MdWRKY33 expression. **b** Luciferase activity assay. **c** MdERF1 and MdERF2 expression. **d** Identified or putative elements in the employed promoters (ref. S1-S4)

**Fig. S3a** Protein expression in apple protoplasts isolated from callus cells cultured for different times. **b** Protein accumulation in apple protoplast cells after transfection for different time. The protoplast cells were isolated from callus cells cultured for 6 days

**Fig. S3a** Protein expression in apple protoplasts isolated from callus cells cultured for different times. **b** Protein accumulation in apple protoplast cells after transfection for different time. The protoplast cells were isolated from callus cells cultured for 6 days


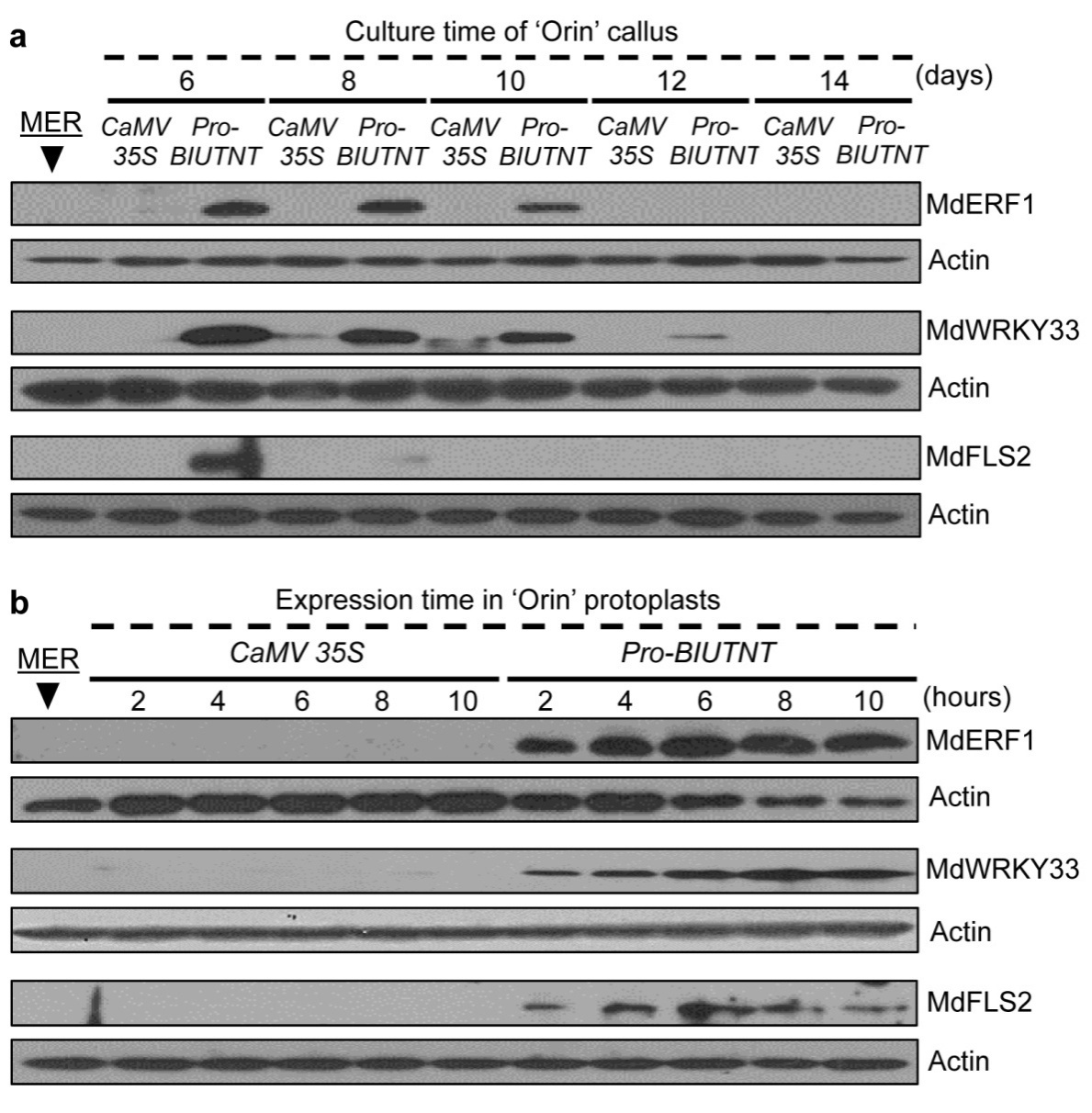


**Fig. S3 a** Protein expression in apple protoplasts isolated from callus cells cultured for different durations. **b** Protein accumulation in apple protoplast cells after transfection for different durations. The protoplast cells were isolated from callus cells that had been cultured for 6 days


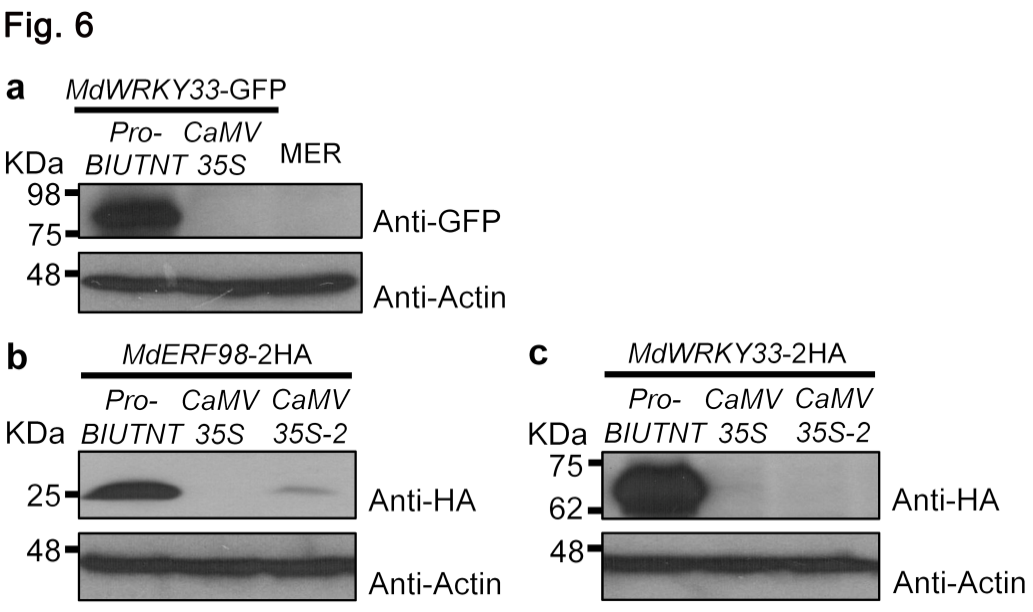


**Fig. S4 Further comparison of *CaMV 35S* and *Pro*-*BIUTNT* in driving the expression of target genes in apple protoplasts. a** MdWRKY33-GFP expression driven by *Pro-BIUTNT* and *CaMV 35S*. **b, c** Comparison of *Pro-BIUTNT* with another variant of the *35S* promoter, *CaMV 35S-2*, in driving MdERF98 (b) and MdWRKY33 (c) expression


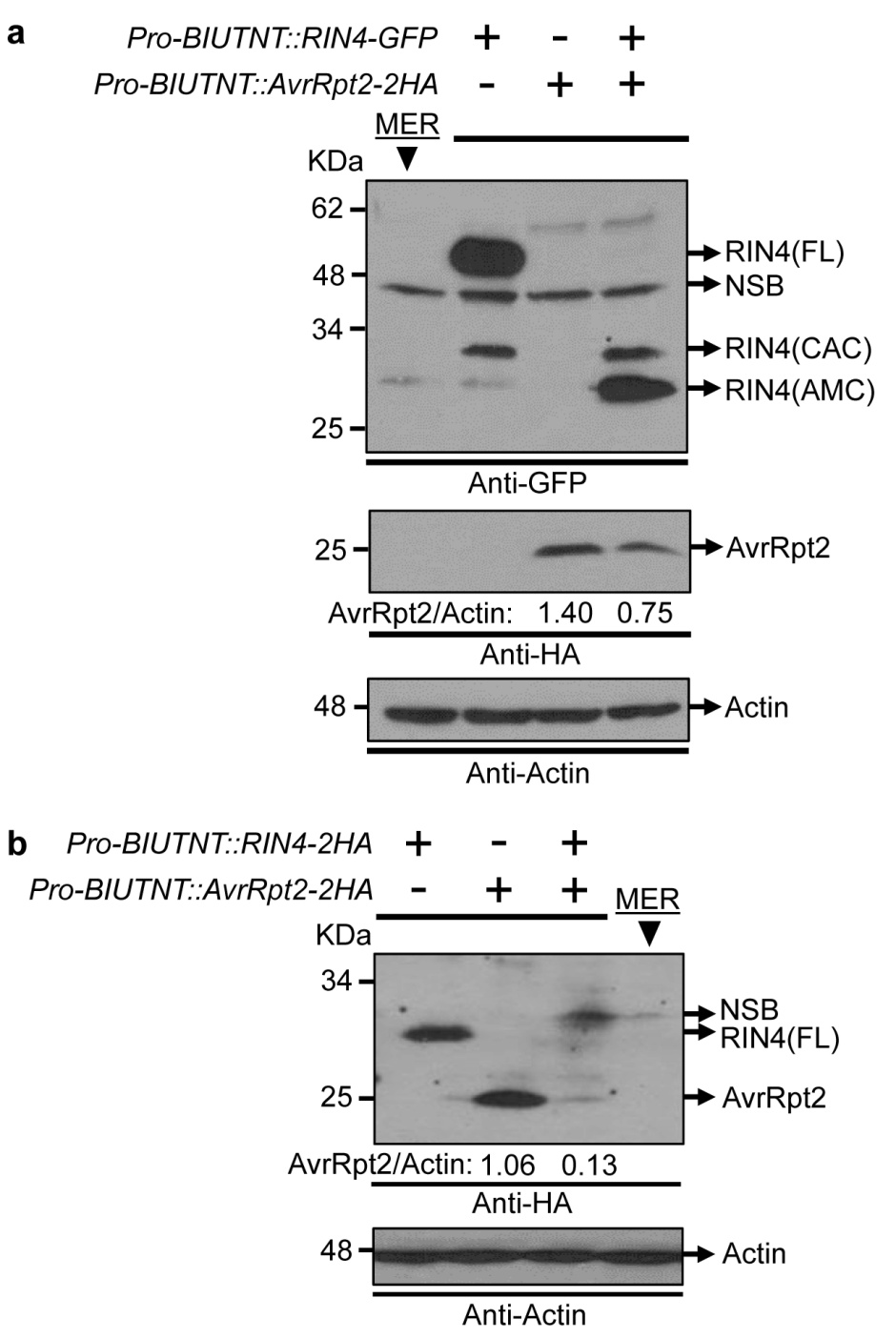


**Fig. S5 Repeat assay of AvrRpt2-mediated RIN4 cleavage. a** The complete repeat as shown in Fig. 6d. **b** The cleavage of RIN4 by AvrRpt2 and the instability of AvrRpt2 in the presence of RIN4. Both RIN4 and AvrRpt2 were tagged with HA at the C-terminus

**Fig. S6 Promoter activity in *Arabidopsis* and apple protoplast cells.** Both *CaMV 35S* and *Pro-BIUTNT* were fused with luciferase and transfected into *Arabidopsis* and apple protoplasts. After expression for 6 h, the luciferase activity was measured as described in the Materials and methods. The asterisks indicate a significant difference (**p<0.01)


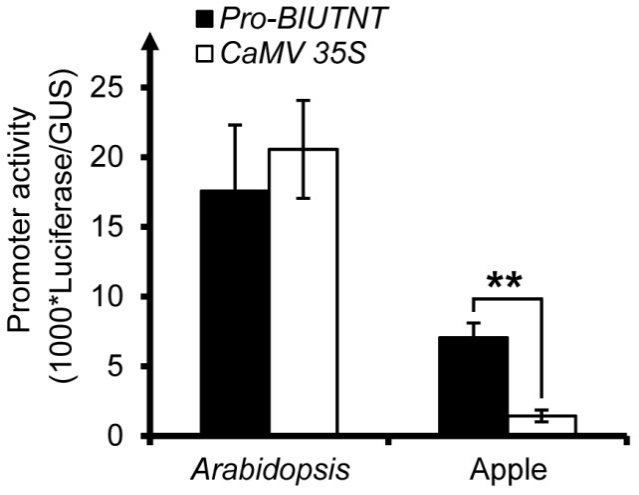


**Sequence 1**

***CaMV 35S***

AAGCTTACTCCAAGAATATCAAAGATACAGTCTCAGAAGACCAAAGGGCTATTGAGACTTTTCAACAAAGGGTAATATCGGGAAACCTCCTCGGATTCCATTGCCCAGCTATCTGTCACTTCATCAAAAGGACAGTAGAAAAGGAAGGTGGCACCTACAAATGCCATCATTGCGATAAAGGAAAGGCTATCGTTCAAGATGCCTCTGCCGACAGTGGTCCCAAAGATGGACCCCCACCCACAAGGAGCATCGTGGAAAAAGAAGACGTTCCAACCACGTCTTCAAAGCAAGTGGATTGATGTGATATCTCCACTGACGTAAGGGATGACGCACAATCCCACTATCCTTCGCCCCAAGCTTGGGCCCAAGCTTGGGTCGCGCCCCACGGATGGTATAAGAATAAAGGCATTCCGCGTGCAGGATTCACCCGTTCGCCTCTCACCTTTTCGCTGTACTCTCTCGCCACACACACCCCCTCTCCAGCTCCGTTGGAGCTCCGGACAGCAGCAGGCGCGGGGCGGTCACGTAGTAAGCAGCTCTCGGCTCCCTCTCCCCTTGCTCCGT

**Sequence 2**

***CaMV 35S-2***

GTCCCCAGATTAGCCTTTTCAATTTCAGAAAGAATGCTAACCCACAGATGGTTAGAGAGGCTTACGCAGCAGGTCTCATCAAGACGATCTACCCGAGCAATAATCTCCAGGAAATCAAATACCTTCCCAAGAAGGTTAAAGATGCAGTCAAAAGATTCAGGACTAACTGCATCAAGAACACAGAGAAAGATATATTTCTCAAGATCAGAAGTACTATTCCAGTATGGACGATTCAAGGCTTGCTTCACAAACCAAGGCAAGTAATAGAGATTGGAGTCTCTAAAAAGGTAGTTCCCACTGAATCAAAGGCCATGGAGTCAAAGATTCAAATAGAGGACCTAACAGAACTCGCCGTAAAGACTGGCGAACAGTTCATACAGAGTCTCTTACGACTCAATGACAAGAAGAAAATCTTCGTCAACATGGTGGAGCACGACACACTTGTCTACTCCAAAAATATCAAAGATACAGTCTCAGAAGACCAAAGGGCAATTGAGACTTTTCAACAAAGGGTAATATCCGGAAACCTCCTCGGATTCCATTGCCCAGCTATCTGTCACTTTATTGTGAAGATAGTGGAAAAGGAAGGTGGCTCCTACAAATGCCATCATTGCGATAAAGGAAAGGCCATCGTTGAAGATGCCTCTGCCGACAGTGGTCCCAAAGATGGACCCCCACCCACGAGGAGCATCGTGGAAAAAGAAGACGTTCCAACCACGTCTTCAAAGCAAGTGGATTGATGTGATATCTCCACTGACGTAAGGGATGACGCACAATCCCACTATCCTTCGCAAGACCCTTCCTCTATATAAGGAAGTTCATTTCATTTGGAGAGAACACGGGGGAC

The underlined regions denote the core sequence of *CaMV 35S* promoter (ref. S2)

**References**

1. Hamilton, D.A., Schwarz, Y.H. & Mascarenhas, J.P. A monocot pollen-specific promoter contains separable pollen-specific and quantitative elements. *Plant Mol. Biol.* **38**, 663–669(1998).
2. Fang, R.X., Nagy, F., Sivasubramaniam, S. & Chua, N.H. Multiple cis regulatory elements for maximal expression of the cauliflower mosaic virus 35S promoter in transgenic plants. *Plant Cell* **1**, 141–150(1989).
3. Gallegos, J.E. & Rose, A.B. An intron-derived motif strongly increases gene expression from transcribed sequences through a splicing independent mechanism in *Arabidopsis thaliana*. *Sci. Rep.* **9**, 1–9(2019).
4. Rose, A.B. The effect of intron location on intron-mediated enhancement of gene expression in *Arabidopsis*. *Plant J.* **40**, 744–751(2004).
